# Using Qualitative Methods to Understand Physical Activity and Weight Management Among Bangladeshis in New York City, 2013

**DOI:** 10.5888/pcd13.160077

**Published:** 2016-07-07

**Authors:** Lindsey Riley, Saima Mili, Chau Trinh-Shevrin, Nadia Islam

**Affiliations:** Author Affiliations: Lindsey Riley, The Michael J. Fox Foundation, New York, New York; Saima Mili, Chau Trinh-Shevrin, New York University, School of Medicine, Department of Population Health, New York, New York.

## Abstract

**Introduction:**

South Asians experience high rates of cardiovascular disease and type 2 diabetes, coupled with low rates of reported physical activity. We report findings from a qualitative sub-study that was conducted in 2013 among Bangladeshi immigrants in New York City to understand factors that affect physical activity practices and weight management in this community.

**Methods:**

Qualitative study participants were recruited from community-based settings. Sex-specific focus groups were conducted by trained community health workers. Proceedings were audio-recorded for translation and transcription and coded using a constant comparative approach. Data were coded using Atlas.ti software.

**Results:**

Six focus groups were completed with a final sample of 67 participants (63% male, 37% female). Mean participant age was 42 years; mean years of residence in the United States was 12. Key themes that emerged were beliefs about modesty and sex-separated facilities that may prevent women from engaging in physical activity. Distinctions were made between men and women about what constitutes exercise versus physical activity; religious prayer was considered to be health-promoting because of the movement involved. Other important themes that emerged were cultural dietary practices and evolving conceptions of healthy weight.

**Conclusion:**

Tailored interventions that take into account the cultural context of this growing community are needed. Findings may also provide insight into barriers to health promotion experienced by other US Muslim communities, which are growing rapidly.

## Introduction

Although physical activity can help prevent or delay the onset of obesity, only 1 in 5 US adults meet the Centers for Disease Control and Prevention’s (CDC’s) 2008 Physical Activity Guidelines (1). Racial and ethnic minorities, including those in immigrant communities, report lower rates of physical activity than do non-Hispanic white adults (2). People from South Asia (ie, India, Pakistan, and Bangladesh) have lower rates of physical activity than the general population and than other racial/ethnic groups (3,4). A limited number of studies have documented low rates of physical activity among South Asian populations overall in the United States (5–7).

South Asians also experience high rates of cardiovascular disease (CVD) and type 2 diabetes (T2D), although certain South Asian subgroups may be at particular risk (8–13). For example, Bangladesh has the youngest mean age of myocardial infarction among all South Asian countries (12). Similarly, studies in the United Kingdom found that Bangladeshi immigrants had more coronary heart disease risk factors compared with the general population (14) and were twice as likely to have diagnosed T2D (15).

As of 2010, more than 277,000 Bangladeshis resided in the United States, making this community the fastest-growing Asian American subgroup in the country (16). In 2013, the largest enclave of Bangladeshis in the United States resided in New York City where 53% of the community had limited English proficiency and 33% lived below the poverty line (17). However, despite the high prevalence of CVD and diabetes-related risk factors among Bangladeshis in Bangladesh and other countries, few studies have examined the chronic disease management practices and health behaviors of Bangladeshis in the United States (18).

In 2010, the DREAM (Diabetes Research, Action, and Education for Minorities) Project study was initiated to evaluate the efficacy of a 6-month community health worker (CHW) program to improve diabetes control and management among Bangladeshis living in New York City (18–20). Preliminary baseline findings from the DREAM Project study highlighted low levels of self-reported physical activity among study respondents, coupled with high levels of self-reported stress. The purpose of our study was to report findings from a qualitative sub-study conducted in 2013 among Bangladeshi immigrants in New York City to understand factors that affect physical activity and weight management in this community.

## Methods

CHWs trained to moderate focus groups and administer surveys conducted 6 focus groups with adult women and men (aged 18–75 years) living in New York City who self-identified as Bangladeshi. Study participants were purposively recruited from community-based settings using word of mouth and snowball sampling methods. Focus group participants were asked about perceptions of weight management, physical activity, social stressors, and acceptable strategies for health promotion. All focus groups were sex-segregated and conducted by a sex-concordant CHW. Human subjects research approval for the study was obtained from the New York University School of Medicine institutional review board; all participants provided written consent before study participation.

Focus group discussions were digitally recorded for later transcription and translation into English. A codebook was developed that used the moderator guide as an initial outline for primary codes, and secondary and tertiary codes were developed throughout the analysis process. Three independent coders who were fluent in Bangla reviewed and analyzed the transcripts through direct content analysis by using a constant comparative approach (21). ATLAS.ti 6 (Scientific Software Development GmbH) was used to code and retrieve data. Coding Analysis Toolkit (Texifter, LLC) was used to ensure inter-coder reliability among study investigators.

## Results

Mean participant age was 42 years; mean years of residence in the US was 12 (Table 1). Sixty-three percent of participants reported a current diagnosis of T2D, and 64% reported a family history of T2D; 66% reported engaging in physical activity within the last 7 days. However only 57% met recommended weekly physical activity levels.

**Table 1:** Demographic Characteristics, Bangladeshi Focus Group Participants (N = 67), New York City, 2013

Characteristic	Men (n = 41), n (%)	Women (n = 25), n (%)
**Age, y**		
18–40	18 (44)	12 (48)
41–60	16 (39)	13 (52)
61–75	7 (17)	0
**Years residing in United States**		
0–10	18 (44)	15 (60)
11–20	14 (34)	6 (24)
≥21	9 (22)	4 (16)
**Self-reported diagnosis of type 2 diabetes**		
Yes	16 (39)	7 (28)
No	25 (61)	18 (72)
**Meets physical activity recommendations**		
Yes	23 (56)	15 (60)
No	18 (44)	10 (40)
**Family history of . . .^a^**		
Type 2 diabetes	26 (63)	17 (68)
Hypertension	24 (58)	19 (76)
Heart disease	13 (32)	10 (40)
Stroke	8 (19)	11 (44)

a Participants could choose more than one response.

### Physical activity

#### Religious and cultural beliefs as both barriers to and facilitators of physical activity

We summarized key themes from the focus groups and implications for the parent intervention in Table 2. Focus group respondents cited cultural and religious beliefs in the Bangladeshi community, a predominantly Muslim population, as a key barrier to participating in physical activity. Both men and women noted that it was deemed culturally inappropriate for women to engage in physical activity openly and in public settings. Respondents also cited a lack of available resources for physical activity for women in the community while remaining in accordance with cultural and religious norms. Facilities or programs in which both men and women engaged in physical activity at the same time were viewed as acceptable only for “foreigners” (ie, non-Muslims or Bangladeshi Americans):

**Table 2:** Key Themes From Bangladeshi Focus Groups and Implications for Parent Intervention, New York City 2013

Theme	Example Quotations	Implications for Community Health Worker Intervention
**Physical activity**		
1) Lack of culturally appropriate resources	“Actually I think that for a few related reasons [I] cannot go to the gym because sometimes it can be seen that there is no gym for only women that we can’t find. That’s why we can’t go. And plus because our country is Muslim, going outside and go running, these things we cannot do. Exercising that way doesn’t happen.” [Female respondent] “So many women they cannot go outside and walk like freely like other cultures . . . that’s the one big problem.” [Male respondent]	Development of a culturally appropriate exercise DVD for Bangladeshi women, incorporating holistic conceptualization of health held by South Asian cultures
2) Religious activity as form of exercise	“Praying standing up is exercise. Sitting and praying is not exercise.” [Female respondent] “First, I offer my prayer five times a day and walk to mosque and back . . . and every Monday and Wednesday I keep my fast during the day weekly two days . . . this falls under exercise for me . . . ” [Male respondent]	Create intervention curriculum content that addresses prayer as physical activity and not exercise (see below)
3) Gender differences in the conception of physical activity vs structured exercise	“Well I drive a taxi, and when I go to airport, I do rope jumping . . . it’s the best to stay young, and I can do 100 at a time . . . ” [Male respondent]	Create intervention curriculum content to differentiate the concepts of physical activity and exercise, and underscore the value and importance of both
	“I also think it falls under exercise because [I] have to run after my children, feed them, and my whole body is moving around. Exercise means moving around body and keeping active . . . ” [Female respondent]	
	“Regarding exercise, I do chores at home. Housework, I do all of it, like washing clothes, organizing, cooking, work that is in the house, and that’s it. Other than that, I don’t do anything else.” [Female respondent]	
	“I exercise. I cook and clean at home. Take kids to and from school.” [Female respondent]	
**Weight management**		
1) Aligning cultural dietary practices with generally promoted dietary practices	“I think that as Bengalis, rice for us is fast food. Rice, meat, and fish. We, at least once a day, eat. . . . We do not eat appropriate portions. However long we are not satisfied, we eat more rice.” [Male respondent]	Incorporate healthier modifications of traditional diet into nutrition curriculum content (eg, brown rice instead of white rice, whole wheat flour for chappatis/naan breads, baking instead of frying, healthier oils in lieu of ghee, etc)
	“If we don’t eat rice for one meal, then we feel like we didn’t eat anything. Even if we eat other things, if we don’t eat rice then we feel like we didn’t eat anything. After the whole day, and then at night, we eat rice.” [Female respondent]	
2) Evolving cultural conceptions of healthy weight	“I wanted to say that being fat no one likes it. Whether it is in Bangladesh or in America, no one likes it. Everyone likes healthy, but no one likes fat.” [Female respondent]	Greater awareness for the evolving social norms around weight and body image
	“Being fat is beautiful, but now, no one likes it. If you are fat, then wearing clothes feels bad. No one says good things.” [Female respondent]	
	“. . . Especially in the village area. They will say that she is living peacefully. She became fat. Food, in her house there is no lacking. Us Bengalis, that’s what we think. That there’s no lacking in their household. They are living very happily and peacefully. Her husband loves her a lot. She is eating and becoming fat.” [Male respondent]	
	“Forty or 50 years ago way back maybe they would say, now however, it is different, people are health concerned, they know it’s not a sign of richness or anything, getting fat.” [Male respondent]	

Abbreviation: DVD, digital versatile disc.

Female respondent: No, we are Muslim. For us, remaining inside covering, doing whatever [activity] is needed. Following foreigners [Americans], it is not possible for us to exercise like them.

Female respondent 1: [For us] there has to be separate accommodations.

Moderator: Men and women?

Female respondent 1: Yes, yes. Separately, separately.

Female respondent 2: We are Muslims.

Respondents noted that this barrier could be mitigated by culturally appropriate sex-segregated physical activity classes: “For example, we exercise at WSP [community-based organization], a woman teaches us, we are all women who do this, there is no reason for these things to be questioned.” (Female respondent)

However, many respondents noted that the availability of culturally appropriate resources were limited or that similar cultural and religious norms applied to outdoor facilities, such as parks, prohibiting women from engaging in structured exercise such as running or walking groups in these settings. Home-based fitness equipment (referred to as “instruments”) was cited as a potential option for women who do not otherwise have access to culturally appropriate resources. A similar preference for private physical activity accommodations was also cited by older men. These types of equipment were, however, viewed as expensive and often too loud or intrusive (eg, treadmills) for individuals living in urban settings.

#### Religious activity as physical activity

Although religious and cultural notions of the appropriateness of engaging in physical activity in public settings were cited as a barrier to health promotion, many respondents conceptualized religious activity itself as an important source of physical activity, specifically the act of *namaz* (ritual Muslim prayer), which involves a series of posturing that incorporates bowing, standing, kneeling, and prostrating. “God gave us [this] system of prayer to keep us well.” (Male respondent)

Because prayers typically occur 5 times per day and may involve walking to a mosque, several respondents noted that this was a sufficient form of exercise. Ritual fasting in accordance with religious practice was also viewed as a health-promoting behavior:

. . . [I]f we lower the amount of food we eat, then I feel I am very well, I am less tired, and after few days I can see I lost some weight. Like during the month of Ramadan, I felt great that I lost 10 to 15 pounds. However, after the end of Ramadan, eating habits go back to previous norms . . . (Male respondent)

#### Conceptions of exercise versus physical activity

A key difference that emerged between male and female focus group respondents was the conception of physical activity versus exercise. Women overwhelmingly viewed the tasks associated with being a homemaker as exercise, frequently citing specific physical benefits gained from their domestic responsibilities:

If you wash the house floors, if you use a broom and clean, then [you] feel pressure on stomach and the stomach benefits. (Female respondent)

However, men were more likely to classify these activities as required daily activity (but not exercise).

Male respondent 1: Not really actually, [women] do activities, cooking and stuff, a lot of the times they are running around . . .

Male respondent 2: It’s not — exercise and activity are different.

Several generational differences in the conception of physical activity versus exercise also emerged. Younger men were more likely to report engaging in group-based sports, such as soccer or cricket, and were also more likely to use gymnasium facilities for structured exercise. Conversely, older men were also more likely to classify occupational activity as a form of exercise:

Actually the job I do, this is like my gym, but I do not do regular gym activities, because I can’t cover it with my time. My mentality is such that if I can’t find designated time for gym . . . I can’t do that but my work is my gym.” (Male respondent)

Younger men, however, were more likely to differentiate between occupational activity and exercise:

Many people think, “Oh I work very hard, I have a difficult job, so exercise is not critical for me.” But actually it’s not so. Exercise is a different thing, and work is a different thing. (Male respondent)

Men also noted the distinction between sedentary occupations (such as taxi driving) and those that generally required more activity throughout the day as having different impacts on weight and general health: “I just want to add, working depends on what kind of work like [he] said, if you do construction work, construction consumes a lot of energy.” (Male respondent)

Both men and women frequently mentioned walking as an acceptable form of exercise. However, male respondents were more likely to note the distinction between walking as a means of transportation in an urban setting and walking for the purposes of exercise.

If you walk in jogging style, then it is exercise. (Male respondent)

Regular walking and exercise walking are different. (Male respondent)

### Weight management

#### Cultural dietary practices

The juxtaposition of commonly prescribed dietary modifications with cultural food preferences was discussed by many focus group respondents as a barrier to managing a healthy weight. In particular, respondents frequently underscored the importance of rice as an important dietary staple and that eliminating or reducing rice consumption would not be feasible:

If we don’t eat rice for one meal, then we feel like we didn’t eat anything. Even if we eat other things, if we don’t eat rice then we feel like we didn’t eat anything. After the whole day, and then at night, we eat rice. (Female respondent)

Regarding Bengali foods, rice and fish, this is . . . this is for us pure. The right amounts, we all know that eating small amount of rice at night is best, but because we love it, we cannot get rid of it. (Male respondent)

If I think about my own weight, [I] control carbohydrates. But I cannot get rid of rice completely, because [I am] used to it, from birth. (Female respondent)

#### Evolving conceptions of healthy weight

Focus group respondents expressed varied opinions about obesity and its implications for health and social standing. Some expressed that being overweight or obese has a detrimental impact on health and is perceived negatively by others:

Our tradition, as a Bengali tradition, we eat too much. We are less cautious about our health. Because of this, after eating what we do, this is why we have different diseases that increase, especially when weight increases, including diabetes, other things like heart disease . . . (Male respondent)

Being fat is beautiful, but now, no one likes it. If you are fat, then wearing clothes feels bad. No one says good things. (Female respondent)

Others felt differently, stating that being obese in the country of birth was associated with prosperity and beauty:

. . . Especially in the village area. They will say that she is living peacefully. She became fat. Food, in her house there is no lacking. Us Bengalis, that’s what we think. That there’s no lacking in their household. They are living very happily and peacefully. Her husband loves her a lot. She is eating and becoming fat. (Male respondent)

They say beautiful. In saris or *salwar kameez* (traditional clothing), [they say] “you ate and got fat, and you look very good.” [Female respondent]

## Discussion

We explored the contextual factors affecting physical activity patterns and weight management in the Bangladeshi community of New York City. Our findings are in alignment with those of previous research among Bangladeshi immigrants in the United Kingdom and among other South Asians in the United States, indicating that gender and social norms affect physical activity patterns, particularly for women who are expected to dress modestly and prioritize domestic responsibilities (22–24). Additionally, the lack of facilities and spaces in which women can participate in physical activity in accordance with gender and religious norms is a barrier and suggests that in-home exercise programs may be a more effective health promotion tool for women in this population (22).

Important gender differences in the conception of physical activity versus exercise were predominant among focus group respondents. The finding that women perceived daily activities and domestic work as physical activity is notable and helps to explain why 60% of female respondents reported meeting daily recommendations for physical activity, higher than physical activity rates generally reported in other research in this community (25). Male focus group respondents were more likely to make the distinction between physical activity and exercise, indicating that men are more likely to engage in planned, structured, or repetitive activities with the ultimate objective of improving physical fitness to improve health (26). Our findings suggest that interventions in this community should emphasize the distinction between physical activity and exercise and should underscore the importance of both types of movement as pertinent to health.

Both men and women considered religious activity (*namaz*) as an appropriate form of exercise promoting better health, similar to findings of previous studies that indicate the role that religion plays in the holistic model of health held by this community (18,22,27,28). Current and future interventions should consider the use of health promotion messaging that acknowledges the intersection of both spiritual and physical health, while underscoring the importance and value of increased structured exercise, particularly for Muslim populations.

Although physical activity plays a crucial role in moderating weight management, cultural norms around diet and body size and shape were also highlighted as key themes. Focus group respondents stressed the importance of retaining core elements of a traditional diet, especially rice, and how this often affects attempts at portion control or weight loss. This finding is in alignment with those of previous work and supports the value of culturally tailored strategies when designing or implementing interventions for this community (29,30). Additionally, focus group respondents expressed varying views on what constitutes a healthy weight and how this often is not in alignment with cultural norms around social status and overall well-being. Viewpoints ranged by sex and age, indicating that norms around this construct may be evolving, either as a result of the passage of time or of acculturation processes (22,31). Cultural norms are not static, so public health practitioners should be aware of the evolution of these norms and use flexible approaches when designing programs (32).

This study has several strengths that contribute to robust validity and reliability. The community-based sampling approach used, coupled with the qualitative design, yielded rich contextual data not systematically collected through the parent study or other state and national data sets. Additionally, focus groups were conducted in Bengali by CHWs who were experienced group facilitators and known community leaders, contributing to increased comfort and trust among study respondents and greater internal validity as a result of their unique understanding of cultural nuances. Lastly, focus group proceedings were transcribed and analyzed by native Bengali speakers, which helped to provide valuable context when evaluating the findings. 

Limitations of this study may include selection bias and potential contamination bias, because convenience-sampling methods were used and some participants were not heterogeneous strangers. Efforts to increase internal validity were made through frequent discourse and internal-code agreement checks between study investigators.

The qualitative findings reported are components of a larger community-based participatory research study to address diabetes management by using a CHW approach in the Bangladeshi population. Pilot study results and protocol and methods of the parent study have been previously reported (18–20). In alignment with community-based participatory research principles, the findings from the qualitative study presented here were used in an iterative fashion to enhance the cultural appropriateness of the parent intervention and incorporate evidence-based group and individual level strategies for physical activity promotion (33). 

Qualitative study findings were also used to create a culturally appropriate, in-language exercise DVD for Bangladeshi women. The exercise instruction is led by a Bangladeshi female CHW and incorporates activities such as walking (which presented no challenges to modesty for focus group respondents) and low-impact physical activity appropriate for women with little to no baseline history of participation in structured exercise. Furthermore, the DVD included a brief tutorial on the distinction between physical activity and exercise, framing exercise as “your time” and physical activity as movement associated with fulfilling domestic responsibilities (eg, laundry, groceries). Table 3 demonstrates more fully how findings from the focus groups were used to inform the development of the DVD.

**Table 3:** Recommendations for Content of a Culturally Relevant Physical Activity DVD, Based on Results from Bangladeshi Focus Groups, New York City 2013

Focus Group Finding	DVD Element	DVD Outcome
Religious and cultural beliefs as a barrier toward physical activity	Setting	Production location was chosen and dressed to resemble a home (eg, included a sink, chair, wall tapestry). Additionally, the DVD host used household items such as soup cans or water bottles in lieu of exercise equipment (eg, weights, resistance bands) to demonstrate how viewers can still engage in physical activity without the use of specific fitness equipment.
Religious and cultural beliefs as a barrier toward physical activity	DVD Host–spokesperson(s)	Two female hosts were selected — one older and one younger, one mother and one single, unmarried. Older host was a CHW affiliated with the research and well-known in the community. Additionally, one female appeared in hijab (covering of hair for modesty), and the other remained uncovered, appealing to women of varying degrees of religiosity and age groups.
Conceptions of exercise vs physical activity	Degree of difficulty	Each movement was presented with alternatives to account for those inexperienced in physical activity or with less mobility or flexibility. Additionally, the DVD menu allowed users to elect to complete sections of the DVD rather than the entire content, so that users can progress over time.
Conceptions of exercise vs physical activity; religious activity as physical activity	Cultural and religious norms	DVD instructor(s) made reference to physical activity as specific time taken out of the daily routine to devote to movement of the body. Associations to both religious posturing and cultural references (eg, movement resembling making chappati or bread) were also made by host(s).

Abbreviation: CHW, community health worker; DVD, digital versatile disc.

To our knowledge, our study is the only research on barriers and facilitators of physical activity among Bangladeshi immigrants in the United States. Our study findings are relevant for practitioners, clinicians, and policy makers developing programs for the Bangladeshi community, a rapidly growing population with a high burden of CVD and diabetes. More broadly, our study has implications for the development of culturally tailored programming for other immigrant populations in the United States. Given the rapid growth of the Muslim community in the United States (34), results may provide insight into barriers to health promotion experienced by other Muslim groups that have a cultural context similar to that of Bangladeshi Americans. Furthermore, our findings indicate the need to take the cultural context of women into account in developing health promotion activities, which is relevant given the growing burden of obesity, CVD, and diabetes among racial and ethnic minority women in the United States (35). Research that explores the contextual factors that affect participation in physical activity by diverse communities contributes to a more nuanced understanding of effective methods to prevent obesity.
